# Practitioner Perspectives on the Association Between Mental Fatigue and Injury Risk in High‐Performance Sport: A Mixed Methods Study

**DOI:** 10.1002/ejsc.70028

**Published:** 2025-07-29

**Authors:** Lewis A. Fazackerley, Jack Hickey, Rich D. Johnston, Paul J. Tofari, Ryan G. Timmins, Bart Roelands, Shona L. Halson, Bruno Tassignon, Jo Verschueren, Suzanna Russell

**Affiliations:** ^1^ School of Behavioural and Health Sciences Australian Catholic University Brisbane Australia; ^2^ Sports Performance, Recovery, Injury and New Technologies Research Centre (SPRINT) Faculty of Health Sciences Australian Catholic University Brisbane Australia; ^3^ Department of Sport Science and Nutrition Maynooth University Maynooth Ireland; ^4^ Carnegie Applied Rugby Research Centre (CARR) Institute for Sport Physical Activity and Leisure Leeds Beckett University Leeds UK; ^5^ Human Physiology and Sports Physiotherapy Research Group Faculty of Physical Education and Physiotherapy Vrije Universiteit Brussel Brussels Belgium; ^6^ Faculty of Rehabilitation Sciences REVAL Rehabilitation Research Center Hasselt University Diepenbeek Belgium; ^7^ Performance Services Australian Institute of Sport Bruce Australia; ^8^ Sport Performance Innovation and Knowledge Excellence (SPIKE) Queensland Academy of Sport Nathan Australia

**Keywords:** athletes, cognition, fatigue, qualitative, soft tissue injuries, sports medicine

## Abstract

This study aimed to investigate the perceptions of practitioners involved in injury management within high‐performance sport on the potential interaction between mental fatigue and injury risk, including perceived mechanisms. A sequential explanatory design was used, with phase one implementing a cross‐sectional survey and phase two utilising semi‐structured interviews. An electronic survey of multi‐disciplinary practitioners working in high‐performance sport, specifically invasion‐based team sports, was conducted. Topics included the mechanisms by which mental fatigue may influence risk of injury, potential sex differences, mental fatigue and injury prevention and areas for future research. Preliminary data analysis guided the development of the phase two interview schedule, which aimed to gain a deeper understanding of the association and perceived mechanisms. Forty‐five participants completed the phase one survey, and eight participants completed the phase two semi‐structured interviews. The primary findings of this study suggest that practitioners working in high‐performance sport perceive a link between mental fatigue and risk of injury, primarily acute noncontact injuries. Proposed mechanisms include impaired motor control, poor biomechanics and reduced cognitive function. However, isolating mental fatigue as a direct factor is difficult, due to challenges distinguishing between mental and physical fatigue. The findings of this study indicate practitioners perceive an association between mental fatigue and risk of injury. Future research focused on the mechanisms linking mental fatigue to injury risk is required to empirically examine and determine the validity of this perception. However, athlete management strategies regarding mental fatigue may be incorporated into practice to potentially limit the risk of athlete injury.

## Highlights

- Practitioners perceive that mental fatigue may increase the risk of injuries, in particular noncontact injuries, in high‐performance invasion sport athletes.
- Perceived indicators of mental fatigue which may relate to injury risk include deterioration in athlete posture and technique, decreased focus and poor decision‐making and behavioural changes such as disengagement.
- Practitioners could consider implementing strategies to reduce mental fatigue when indicators are observed, potentially mitigating injury risk. Approaches include brief rest breaks, caffeine‐maltodextrin mouth rinses or exposure to pleasant odours.

## 1 Introduction

Fatigue is a complex and multifaceted phenomenon (Halson 2014), which can be managed by balancing load and recovery to reduce the risk of injury and illness in sports (Soligard et al. 2016). Mental fatigue has emerged as an important aspect of fatigue with potential to impact the risk of injury and illness in sports, as well as human performance (Brown et al. 2020; Loch et al. 2019; Van Cutsem et al. 2017). The effects of mental fatigue on physical, technical, tactical and psychological aspects of human performance have received increased attention over the past decade (Badin et al. 2016; Coutinho et al. 2017; Habay et al. 2021; Van Cutsem et al. 2017). As such, emerging strategies aimed at mitigating the effects of mental fatigue, such as caffeine and the use of specific odours, have recently been proposed (Proost et al. 2022).

Reported impacts of mental fatigue on human performance include impairments in response time, decision‐making and sport‐specific motor performance, evidencing a significant influence on movement patterns (Habay et al. 2021). In addition to the negative impact on human performance, research in healthy adult populations has identified mental fatigue as a risk factor for slips, trips, falls and loss of balance, resulting from changes in biomechanics when placed in compromised situations (Lew and Qu 2014; Qu et al. 2019). Accordingly, mental fatigue could influence injury risk in sports (Schampheleer and Roelands 2024). Despite the potential negative impact and the substantial financial and emotional consequences resulting from sporting injuries (Lutter et al. 2022), the relationship between mental fatigue and injury in athletic populations is yet to be investigated.

The perceptual‐cognitive demands of invasion‐based sports have been highlighted as extremely challenging (Badin et al. 2016). Athletes must remain alert for extended periods, such as 90 to 120+ minutes in football codes, constantly scanning their dynamic performance environment and attending only to relevant information (Mann et al. 2007). Athletes who play invasion‐based team‐sports are likely to experience high levels of mental demand during competition, which is thought to contribute to performance reductions (Van Cutsem et al. 2017). In addition to the acute cognitive demands of competition, periods of congested scheduling have been associated with increased injury risk due to high physical workloads (Dellal et al. 2015). The present authors propose that such scheduling may also impose elevated mental demands, which could further contribute to injury risk. Given the evidence linking mental fatigue to impairments in physical and cognitive performance (Russell et al. 2020; Van Cutsem et al. 2017), as well as alterations in movement patterns (Lew and Qu 2014; Qu et al. 2019), exploratory research studies into potential associations between mental fatigue and sports injury are warranted.

Exploratory research, by means of qualitative methodologies, can offer valuable insights into emerging topics related to sports performance practices, which can inform practitioners (Dietrich and Ehrlenspiel 2010; Harper and McCunn 2017). Utilising sequential techniques, such as a mixed‐methods survey and interview‐based approach, provides valuable information on the current knowledge and practices in applied settings (Edmonds and Kennedy 2016). Given the scarcity of research on mental fatigue and injury, conducting a cross‐sectional survey and using the results to develop semi‐structured interview schedules can provide robust data and facilitate meaningful discussions (Edmonds and Kennedy 2016). Incorporating the perspectives and opinions of practitioners from their observations in daily training and competition settings enables an assessment of the current perceptions of mental fatigue on injury risk. Furthermore, these practitioner perspectives may guide future research to potentially attenuate potential risks of injury during periods where athletes may experience elevations in mental fatigue (Evans et al. 2021).

This study aimed to investigate the knowledge and perceptions of practitioners working in high‐performance sport on potential interactions between mental fatigue and injury risk, types of injury and their mechanisms and directions for future research. Given the absence of research to date, this project utilised a sequential explanatory mixed‐methodology approach to provide novel insights. The findings will aid in the design of future research focused on identifying the underlying mechanisms linking mental fatigue to risk of injury.

## 2 Methodology

### 2.1 Study Design

A mixed‐methods approach with a sequential explanatory design was used. Phase one implemented a cross‐sectional survey design to obtain a broad understanding on knowledge and perceptions on the perceived mechanisms of mental fatigue in relation to injury. Phase two utilised semi‐structured interviews to gain a deeper understanding on the perceived influence and mechanisms of mental fatigue on injury risk. Ethical approval was provided by the university's Human Research Ethics Committee (approval number: 2022‐2742E).

### 2.2 Phase One

#### 2.2.1 Participants

Sports practitioners involved in injury management working with professional invasion‐based team‐sport athletes were invited to participate in this study. Participants were purposefully recruited through social media platforms (X/Twitter and LinkedIn) to facilitate international outreach, alongside direct email communication to national sporting bodies and word‐of‐mouth within professional networks. Eligible participants were required to be practitioners working at a professional or high‐performance level in invasion‐based team sports, with experience in injury management. Relevant eligible practitioner roles included high‐performance managers, physiotherapists, strength and conditioning/physical performance coaches, medical personnel and coaches. All participants provided their informed consent to participate in this study. The athletes whom participants work with were classified according to the high‐performance framework by McKay et al. (2022).

#### 2.2.2 Survey Design

The questionnaire was built with REDCap (Research Electronic Data Capture, USA) and distributed via a generated public survey link. All authors contributed to the development of the research questions and distribution of the public survey link. Following piloting amongst the full research team, a subset of authors (*n* = 4), with specific expertise in mental fatigue, injury and mixed methods designs, refined multiple versions of the survey based on pilot feedback. Alterations included question wording, structure and order. The final survey tool consisted of a three‐point Likert scale (not at all, somewhat and largely), consistent with prior research (Russell et al. 2024), and to simplify response options prior to interviews in phase two. Additionally, visual analogue scales (VAS) and open‐response questions were included. Definitions of mental fatigue and injury/injury type were provided in the survey content to ensure participant understanding and consistency in responses. The survey link was active between March 2023 and May 2024.

#### 2.2.3 Statistical Analysis

Descriptive outputs and quantitative analyses were performed using RStudio (Posit, Boston, Massachusetts, USA) with the R statistical programming language (4.2.2, R Foundation, Vienna, Austria). A one‐way ANOVA (*aov*) was used to assess differences in VAS scores on the perceived relationship between mental fatigue and specific injury types. These injury types were classified firstly based on their mode of onset being either gradual or sudden, with sudden onset injuries sub‐classified based on their mechanism involving either direct contact, indirect contact or noncontact, as per the International Olympic Committee's 2020 consensus statement on reporting of injury data in sports (Bahr et al. 2020). Estimated marginal means (*emmeans*) with a Tukey adjustment were used to determine whether there were differences in the perceived relationship between injury types. Statistical significance was set at *p* < 0.05. The Braun and Clarke (2006) step‐by‐step guide and 15‐point checklist were used to inform the thematic analyses for free text responses.

### 2.3 Phase Two

#### 2.3.1 Participants

On completion of phase one, participants were invited to express their interest to participate in phase two, which include semi‐structured interviews. Participants who accepted the invitation completed an additional consent form via REDCap, and an interview time was scheduled by the lead author.

#### 2.3.2 Topics of Discussion

To establish face validity, topics of discussion were developed by a preliminary analysis of phase one survey results, which were discussed by members of the research team (*n* = 3). Topics of discussion included the mechanisms by which mental fatigue may influence risk of injury, the different types of injuries, potential sex differences in mental fatigue impacting injury risk, how practitioners currently account for mental fatigue in injury prevention and areas for future research. Subsequently, an interview schedule was developed and refined through discussions amongst members of the research team, who hold expertise in mental fatigue and injury. Following a pilot interview (*n* = 1), minor adjustments were made to enhance clarity and delivery. Refinements were aimed to improve face validity, ensuring the questions were clear and aligned with the intended constructs. Definitions of mental fatigue and injury/injury type were provided in the interview preamble to ensure participant understanding and ensure consistency in responses. Semi‐structured interviews were conducted by the lead author and completed between October 2023 and May 2024. The survey remained open during this period, as completion was required for interview invitations. Due to the semi‐structured design of the interview schedule, data saturation was avoided (Braun and Clarke 2021). The full interview schedule can be found in Supporting Information S1.

#### 2.3.3 Interview Process

The semi‐structured interviews were conducted and recorded using Zoom software (Zoom Video Communications Inc., San Jose, California). Immediately prior to the commencement of the interview, a standardised preamble was read verbatim to all participants, which provided clear instructions on the interview process and allowed participants to seek clarification. This ensured that all participants had a consistent comprehension of the interview process to support comparable responses. Following the interview, audio (mp3) was exported from Zoom and transcribed. Unique identifiers were assigned to each participant (e.g., P01, P02) and transcripts sent back to the participants for final approval prior to being exported for data analysis.

#### 2.3.4 Data Analysis

The research team adopted a positivist philosophy, which guided the approach to analysing the data scientifically (Hassmén et al., 2016). Thematic analysis was conducted following the six‐phase process outlined by Braun et al. (2016), which includes familiarisation, coding, theme development, refinement, naming and write‐up. Initially, the lead author immersed themselves in the data by reading each transcript multiple times (typically three to four readings) to ensure thorough familiarity and engagement with the content. Systematic coding using NVivo software (NVivo 12.7, QSR International, Australia) was undertaken, with each relevant segment of data assigned a code representing key concepts. These codes were collated into broader themes during the theme development phase and organised into overarching patterns of meaning. Themes and final outputs were reviewed by LF and SR, who have qualitative research experience, and refined to ensure they accurately represented the data, with clear definitions and names developed for each theme.

## 3 Results

### 3.1 Participants

In phase one, 45 participants (*n* = 36 men, 9 women; age = 38.9 ± 13.2 years) completed the survey. Participants had on average 11.7 ± 8.2 years' experience working in high‐performance sport. A summary of participant characteristics can be viewed in Table 1. Phase two included eight participants (*n* = 7 men, 1 woman), who participated in a semi‐structured interview. Six participants were employed in Australia, and two participants were employed in Belgium. Participants involved in the semi‐structured interviews had extensive experience across a large range of high‐performance sport roles, with their current primary role in strength and conditioning (*n* = 3), physiotherapy (*n* = 2), head coaching (*n* = 1), head of medical (*n* = 1) and sport science data analysis (*n* = 1). All eight participants in Phase 2 reported experience working with professional, elite, or highly trained athletes competing at national or international levels. The interviews ran for an average duration of 36.5 ± 8.7 min.

**TABLE 1:** Summary of key participant demographics in phase one.

Demographic	*n* (% of respondents)
Location	
Australia	30 (66.7)
Ireland	5 (11.1)
United States of America	5 (11.1)
Belgium	3 (6.7)
United Kingdom	1 (2.2)
Germany	1 (2.2)
Highest qualification	
Masters (coursework)	18 (40.0)
Masters (research)	10 (22.2)
Bachelor’s degree	9 (20.0)
Doctor of philosophy	6 (13.3)
Honours	1 (2.2)
Certificate	1 (2.2)
Organisation type/s *	
Professional/elite Club or team	30 (66.7)
Highly trained/national level	25 (55.6)
Elite/international level	22 (48.9)
National or state/area institute	16 (35.6)
World class	16 (35.6)
Trained/developmental	11 (24.4)
Semi‐professional/semi‐elite club or team	9 (20.0)
National sporting organisation	7 (15.6)
Recreationally active	3 (6.7)
Primary sport/s *	
Australian rules football	18 (40.0)
Soccer	13 (28.9)
Rugby league/union	8 (17.8)
Basketball	6 (13.3)
Gaelic football	6 (13.3)
Hurling	3 (6.7)
*Other*	22 (48.9)
Role/s *	
Strength and conditioning/physical performance coach	18 (40.0)
Physiotherapist/physical therapist	15 (33.3)
Sports scientist	9 (20.0)
High performance manager	7 (15.6)
Athletic trainer/physical trainer	3 (6.7)
Exercise physiologist	2 (4.4)
Head coach	2 (4.4)
Medical doctor	1 (2.2)
Head of medical	1 (2.2)
Sex (at birth) of athletes coached	
Male	25 (55.6)
Female	13 (28.9)
Both male and female	6 (13.3)

*Note:* Headings with an asterisk (*) were questions where participants could choose multiple responses.

### 3.2 Phase One

**FIGURE 1:**
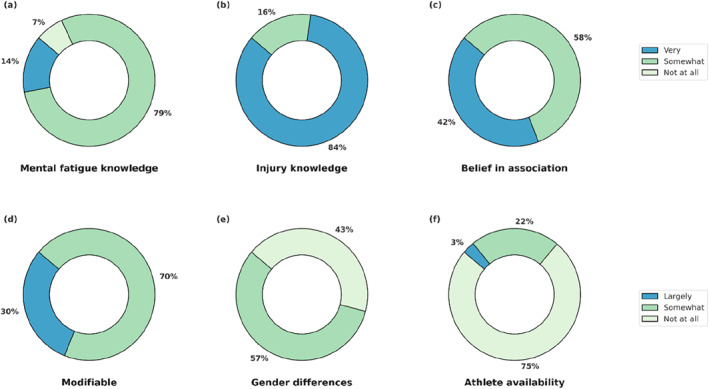
Distribution of responses on the knowledge of mental fatigue (a) and injury (b), perceptions on the association between mental fatigue and injury (c), whether the mechanisms are modifiable (d), whether the association is different between genders (e) and whether mental fatigue affects athlete availability beyond injury (f).

Figure 1 presents perceived knowledge on mental fatigue and injury, as well as perceptions about the association between mental fatigue and injury. It also presents perceptions on whether these associations are modifiable, vary by gender or influence athlete availability. Table 2 summarises free‐text responses for these topics.

**TABLE 2:** Summary of thematic analysis on the modifiability of mechanisms and mitigation strategies, gender differences and athlete availability practice.

Topic	Description
Modifiable/mitigation strategies	Adjusting training loads and regular monitoring are key strategies to reduce injury risk under mental fatigue (*n* = 14, 28%).
	Providing psychological support and using recovery techniques are crucial in managing mental fatigue (*n* = 10, 20%).
	Improving sleep hygiene and ensuring proper nutrition are essential strategies to combat mental fatigue (*n* = 9, 18%).
	Mental training and cognitive load periodisation help athletes handle stress and fatigue more effectively (*n* = 8, 16%).
	Minimising athletes' time at the club and managing schedules can effectively reduce mental fatigue (*n* = 6, 12%).
Athlete availability	Mental fatigue affects an athlete's performance and readiness, potentially leading to reduced availability (*n* = 11, 24%).
	Mental fatigue is linked to a higher risk of illness, which can reduce athlete availability (*n* = 10, 22%).
	Mental fatigue can cause athletes to withdraw from training sessions or competitions, leading to decreased availability (*n* = 9, 20%).
	Mental fatigue may contribute to or exacerbate mental health issues, affecting an athlete's availability (*n* = 6, 12%)
Gender difference	Uncertainty or lack of knowledge regarding gender differences in mental fatigue (*n* = 11, 33%).
	Hormonal fluctuations, particularly related to the menstrual cycle, were frequently mentioned as potential reasons for gender differences in mental fatigue (*n* = 6, 18%).
	There are no significant gender differences in how mental fatigue affects athletes (*n* = 6, 18%).
	Female athletes may experience greater mental fatigue due to additional life stressors, such as balancing work, family and sport (*n* = 5, 15%).
	Personality traits, such as anxiety, which might be more prevalent in females, could influence mental fatigue (*n* = 4, 12%).

*Note:* Number of participants and percent respondents contributing to each theme (*n*, %).

**FIGURE 2:**
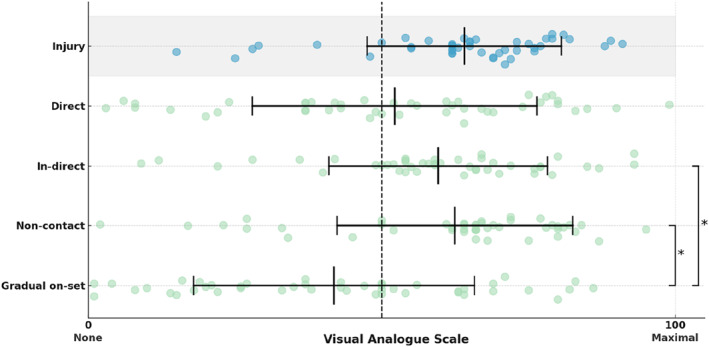
Visual analogue scale responses showing the mean ± SD of the perceived strength of the association between mental fatigue and injury (grey) and for specific injury types. An asterisk (*) denotes injury types that are significantly different from one another.

When asked about the strength of the perceived association between injury risk and mental fatigue on a VAS, with the scale anchored by none at all (0) to maximal (100), respondents indicated an average association of 64.1% (Figure 2). Regarding specific injury types, respondents estimated the strength of the perceived association between mental fatigue and direct contact injuries at 52.2%, indirect contact injuries at 59.6%, noncontact injuries at 62.5% and gradual onset injuries at 41.9% (Figure 2). When comparing different injury types, there was a statistically significant interaction with mental fatigue (*p* < 0.001). Post hoc analysis revealed that the perceived association between mental fatigue and gradual onset injuries was significantly lower compared to both indirect contact injuries (*p* = 0.001) and noncontact injuries (*p* < 0.001). Table 3 summarises free text responses regarding the association between mental fatigue and general injury risk and then the specific injury types.

**TABLE 3:** Summary of thematic analysis on the mechanisms between mental fatigue and injury type.

Injury	Theme	Description
General association	Impaired decision‐making and cognitive function	Mental fatigue leads to poor decision‐making, slower reaction times and cognitive impairment, increasing injury risk (*n* = 12, 31%).
	Impact on physical performance and motor control	Mental fatigue reduces motor control, coordination and movement quality, elevating the likelihood of injury (*n* = 10, 26%).
	Interaction with other factors	Mental fatigue contributes to injury risk alongside other factors like physical fatigue, stress and sleep deprivation (*n* = 9, 23%).
	Psychological and emotional stress	Psychological stress and mental fatigue increase injury risk, particularly during high‐stress periods or personal challenges (*n* = 8, 20%).
Direct contact	Impaired decision‐making and reaction time	Mental fatigue leads to poor decision‐making and slower reaction times, increasing injury risk (*n* = 17, 45%).
	Biomechanical and physical factors	Direct injuries result from biomechanical factors, with mental fatigue possibly influencing but not directly causing (*n* = 10, 26%).
	Limited association or scepticism	Scepticism about mental fatigue's influence on direct contact injuries, with other factors seen as more impactful (*n* = 7, 18%).
	Influence of mental fatigue on contact injury outcomes	Mental fatigue influences the outcome of contact injuries by affecting the body's response to force (*n* = 5, 13%).
Indirect contact	Impaired decision‐making and coordination	Mental fatigue leads to impaired decision‐making, reduced coordination and slower reaction times (*n* = 16, 47%).
	Influence of mental fatigue on injury risk	Mental fatigue plays a role in increasing injury likelihood, but is not the sole determinant (*n* = 8, 22%).
	Limited association or scepticism	Scepticism about mental fatigue's impact on indirect contact injuries, considering other factors more influential (*n* = 6, 17%).
	Biomechanical and physical factors	Indirect injuries are influenced by multiple factors, where mental fatigue may play a secondary role (*n* = 5, 14%).
Non‐contact	Impaired decision‐making and cognitive function	Mental fatigue leads to cognitive impairment, resulting in poor decision‐making and increased injury risk (*n* = 13, 36%).
	Decreased motor control and coordination	Mental fatigue reduces motor control and coordination, leading to poor movement patterns and increased injury risk (*n* = 10, 28%).
	Limited association or scepticism	Scepticism about the extent of mental fatigue's influence on noncontact injuries, with other factors playing a role (*n* = 7, 20%).
	Influence of mental fatigue on physical readiness	Mental fatigue affects physical readiness, contributing to noncontact injuries (*n* = 6, 17%).
Gradual onset	Impaired recovery and chronic stress	Mental fatigue impairs recovery, disrupts hormonal balance and contributes to chronic stress, increasing injury risk (*n* = 10, 29%).
	Influence on movement patterns and biomechanics	Mental fatigue affects movement patterns and biomechanics over time, leading to gradual onset injuries (*n* = 9, 27%).
	Limited association or scepticism	Scepticism about mental fatigue's impact on gradual onset injuries, with other factors seen as more influential (*n* = 8, 24%).
	Contribution to overuse and accumulation of stress	Mental fatigue contributes to overuse injuries by affecting load management and leading to the accumulation of stress (*n* = 7, 20%).

*Note:* Number of participants and percent respondents contributing to each theme (*n*, %).

**FIGURE 3:**
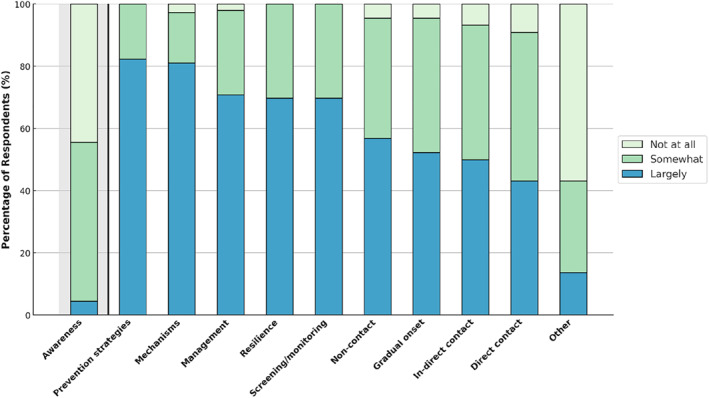
Distribution of respondents on the extent to which evidence from future research on these topics would inform their practice with athletes. ‘Awareness' indicates respondents’ familiarity with existing scientific literature on mental fatigue, mental recovery and injury within athletic populations.

Figure 3 presents the distribution of respondents' perceptions on 11 aspects of mental fatigue and injury risk that would benefit from future research. Participants who suggested ‘other’ had the opportunity to propose topics for future research which included effective methods for measuring and monitoring mental fatigue in athletes (*n* = 6), exploring specific causes of mental fatigue and differences between athletes (*n* = 3), investigating the relationship between mental fatigue, performance and injury (*n* = 3) and strategies for mitigating mental fatigue or integrating mental training to reduce its impact (*n* = 2).

### 3.3 Phase Two

#### 3.3.1 Perceived Association and Mechanisms of Mental Fatigue and Injury Risk

Participants in phase two highlighted a perceived association between mental fatigue and an increased risk of injury, identifying various mechanisms through which this relationship may occur. One of the main mechanisms discussed was the link between mental fatigue and noncontact injuries. Five participants (62.5%) suggested that mental fatigue impairs an athlete's ability to execute movements with proper biomechanics, thereby increasing the likelihood of noncontact injuries such as ACL tears or ankle sprains.

I think certainly non‐contact injuries like cruciate ligaments. That’s the biggest injury we see in rugby, and I think non‐contact injuries can be linked with mentally fatigued athletes.

Participant 7

Another frequently mentioned mechanism involved gradual onset injuries. Four participants (50%) discussed that chronic exposure to mental fatigue could lead to gradual onset injuries, such as tendinopathy, due to the cumulative stress placed on the body over time.

If mental fatigue stays for a long term and is too high, then the muscles will react to it gradually. So, the tendinopathy fatigue will gradually increase.

Participant 6

Indirect and direct contact injuries were also discussed, however, less frequently. In the context of indirect contact injuries, four participants (50%) noted that mental fatigue might lower reflexes and cognitive sharpness. Discussion suggests this could make athletes more vulnerable during scenarios that require quick responses, potentially leading to injuries. Regarding direct contact injuries, the discussions were mixed. Three participants (37.5%) suggested that mental fatigue might lead to a lapse in judgement or slower reaction times during physical encounters, potentially leading to injury. However, two participants (25%) were sceptical of a direct link between mental fatigue and contact injuries, emphasising that these injuries are more likely to occur due to physical rather than mental fatigue.

#### 3.3.2 Indicators of Mental Fatigue and Injury Risk

Mental fatigue was linked to observable changes in an athlete's posture and technique, as noted by three participants. They observed that as athletes become mentally fatigued, their physical execution can deteriorate.

When they start to hinge over more when they’re upright or in football terms, their technique is sharp. When they start to get sloppy with their technique or posture, it can be physical but also you see decisions attached to that that are wrong.

Participant 1

Cognitive impairments, such as decreased focus and poor decision‐making, were discussed as signs of mental fatigue by four participants (50%). Mentally fatigued athletes were reported to struggle with tasks requiring high levels of concentration.

When players aren’t moving into position at the right time, it could be showing fatigue. This could also be physical, but the decision‐making elements they get wrong a lot more.

Participant 1

Behavioural changes, such as disengagement from social interactions and a general flatness in mood, were also identified as indicators of mental fatigue by three participants (37.5%).

If you see them either getting by themselves or not really engaging with others. that’s one of the signs.

Participant 4

However, despite these observable indicators, five participants (62.5%) highlighted the difficulty in measuring mental fatigue objectively, with most relying on subjective assessments.

We assess mental fatigue through our self‐reported wellness questionnaires, but from observation alone, there’s nothing that really suggests whether an athlete is mentally fatigued or not.

Participant 8

#### 3.3.3 Impact of Sex on Mental Fatigue and Injury Risk

Two participants (25%) expressed uncertainty regarding sex‐specific differences in the relationship between mental fatigue and injury risk. Three participants (37.5%) did not have the experience with both male and female athletes to provide comments.

I want to say no, but then I also don’t know what I’m basing that off of to be honest.

Participant 4

Three participants (37.5%) perceived that female athletes experience mental fatigue differently than males and that females often focus on maintaining health to perform well. One participant (12.5%) perceived that males prioritise overcoming fatigue to enhance performance. However, these discussions did not directly address the relationship between mental fatigue and injury. Instead, they explored indirect factors like general conditioning, biological differences and cognitive capacity, which may contribute to injury but were not explicitly linked.

Probably one aspect is conditioning. That's not just physical conditioning, but also cognitive capacity. Do they have the capacity to handle the number of decisions or an overload of decisions in a game?

Participant 2

Male and female athletes are exposed to different stimuli across a day and are biologically different. They will respond to stresses differently leading to different responses to mental fatigue.

Participant 8

#### 3.3.4 Impact of Mental Fatigue on Athlete Availability

Participants discussed the influence of mental fatigue on athlete availability, extending beyond its impact on injuries. Four participants (50%) suggested that mental fatigue can reduce an athlete's ability to participate in training and competition.

They come into training tired mentally and physically, affecting participation. We make adjustments or give them the day off.

Participant 1

Conversely, three participants (37.5%) expressed uncertainty about the extent to which mental fatigue alone impacts athlete availability.

I don’t think mental fatigue on its own has led to time‐loss injuries. there are always other factors at play.

Participant 4

#### 3.3.5 Factoring Mental Fatigue Into Training to Mitigate Risk of Injury

Participants shared diverse perspectives on whether and how mental fatigue is deliberately factored into training. Three participants (37.5%) acknowledged that while mental fatigue is not always directly targeted, it is considered within the broader context of training design. This suggests that mental fatigue is managed through strategies aimed at enhancing overall performance.

For me, it’s about performance. It’s less about injury risk. If mental fatigue or reducing mental fatigue helps with that, then I’ve done my job.

Participant 2

We factor in mental fatigue in the timing of injury prevention strategies. On a day they have had significant cognitive and physical stimuli, you would probably do less work because you will lose them mentally.

Participant 3

However, no participants reported deliberately factoring of mental fatigue into their training regimens. This reflects a more traditional focus on physical conditioning, with mental fatigue often being overlooked or managed incidentally rather than deliberately integrated into training protocols.

We only do more physical prevention. But no, we don’t have an aspect for mental fatigue prevention.

Participant 6

#### 3.3.6 Strategies to Mitigate Mental Fatigue to Reduce Risk of Injury

In contrast, there was a broader recognition of the need to mitigate mental fatigue, particularly to enhance athlete well‐being and performance. Five participants (62.5%) described various strategies employed to reduce mental fatigue, ranging from adjusting training schedules to providing psychological support. Adjustments were made to prevent cognitive overload right before a game, thus aiming to keep athletes mentally fresh. However, these were not done specifically in relation to injury, but rather for performance.

We try to do it but it’s really basic. We ask our players if they are mentally fatigued or physically fatigued with just a questionnaire at the beginning of each training camp.

Participant 7

We do the tactical analysis on a Thursday when the game is on a Saturday. because your athletes get mentally fatigued.

Participant 8

#### 3.3.7 Future Research Ideas and Presentation, and Role Responsibilities

All eight participants (100%) identified two key areas for future research. Five participants (62.5%) emphasised the need for objective measures to assess mental fatigue, with a focus on reliable tools beyond self‐reported questionnaires.

It would be good to have an objective measure that’s not just players answering a questionnaire that can indicate mental fatigue.

Participant 3

Three participants (37.5%) highlighted the importance of understanding the correlation between mental fatigue and injury, emphasising the need for evidence to support the role mental fatigue plays in injury risk.

It would be really interesting to look at how much of a role mental fatigue plays in injury. We’re not really thinking about mental fatigue leading to injury.

Participant 4

Seven participants (87.5%) discussed the barriers to addressing mental fatigue in sports, grouping them into time constraints (*n* = 4) and lack of awareness or resistance from athletes (*n* = 3).

The fact that I don’t really know the signs or the background of mental fatigue. That’s holding me back a little bit to implement it.

Participant 6

The battle would be getting the players to consent. If you just presented a battery of tests without understanding that it’s real. They might not want to do it.

Participant 3

All eight participants (100%) expressed a preference for a variety of research presentation formats. Five participants (62.5%) favoured workshops, conferences and seminars as effective methods. Three participants (*n* = 37.5%) preferred more accessible abstracts and publications. All eight participants (100%) discussed who should be responsible for managing mental fatigue, with six (75%) advocating for a multidisciplinary approach, whereas two (25%) suggested defining specific roles within the organisation, thereby emphasising the need for clear responsibility.

Everyone who is surrounding the team. Both the field staff and the medical staff.

Participant 6

## 4 Discussion

The aim of this study was to explore practitioners' knowledge and perceptions on the potential association between mental fatigue and injury risk in invasion sports. To our knowledge, this is the first study to investigate such associations. The primary findings of this study suggest that practitioners working in invasion sports perceive there to be a link between mental fatigue and risk of injury, primarily noncontact injuries. The mechanisms proposed by participants include impaired motor control, poor biomechanics and reduced cognitive function, all of which might contribute to an increased risk of injury. However, distinguishing mental fatigue as an isolated factor is challenging, as it often presents concurrently with physical fatigue, making it difficult to make conclusions regarding its direct impact on injury. The results of this study provide a foundation for future research focused on the physiological mechanisms linking cognitive demands to injury risk.

Findings from both phases of this study suggest that participants working in invasion sports perceive elevated mental fatigue as most likely to increase the risk of noncontact injuries. These results align with the review by Smith et al. (2018), who suggested skilled performance under conditions of mental fatigue may increases the risk of injury. Research in healthy populations further supports this notion, showing that mental fatigue may impair perceptual interpretation of sensory information, leading to poor biomechanics when purposely tripped (Lew and Qu 2014). Therefore, it is postulated that when athletes are mentally fatigued, poor biomechanics could contribute to the risk of noncontact injuries.

In addition to noncontact injuries, participants in phase two expressed a potential relationship with mental fatigue on gradual onset injuries. Interestingly, this was considered less likely to be associated with mental fatigue in phase one, yet still prevalent. In‐depth discussions in phase two and free‐text responses in phase one suggest a perception that chronic exposure to mental fatigue could lead to prolonged stress and biomechanical changes placed on the body over time. Given that physical fatigue and mental fatigue are linked (Marcora et al. 2009), and prolonged states of physical fatigue can lead to overuse injuries (Edwards 2018), there is a theoretical connection that prolonged mental fatigue may result in gradual onset injuries. However, to our knowledge, there is currently no empirical evidence available to support this notion.

Indicators of mental fatigue that may result in injury, identified by participants in phase two, included noticeable physical and cognitive changes. The physical observations are consistent with existing research, which suggests that mental fatigue can result in postural changes, such as increased trunk flexion while walking (Qu et al. 2019). This is also consistent with research that investigated decision‐making under states of mental fatigue, where professional soccer athletes displayed impaired passing decision‐making performance following a 30‐min Stroop task (Gantois et al. 2020). Given that quantifiable and objective indicators of mental fatigue appear to exist in the sporting context and that participants perceive that mental fatigue may result in injury, it is interesting that participants in the present study do not intervene to reduce mental fatigue. However, this may be due to a lack of understanding of mental fatigue, as suggested by responses in phase one. This hesitancy to intervene is consistent with research by Russell et al. (2024), who reported that most practitioners did not assess mental fatigue (61.5%) and do not deliberately manage mental fatigue (55.8%). Loch et al. (2019) suggest that short rest periods in training and competition may allow athletes to quickly recover both mentally and physically. In practice, brief breaks could be introduced on an individual basis (Loch et al., 2019), guided by identified indicators of mental fatigue, such as changes in posture, decreased focus and decision making or disengagement (Russell et al. 2019). Further strategies could also be considered to counter mental fatigue, such as a caffeine‐maltodextrin mouth rinse (Van Cutsem et al., 2018). Alternatively, exposure to pleasant odours (e.g., citral, green and menthol) has been shown to activate brain receptors, which may improve perceptual state and cognition when mentally fatigued (Proost et al. 2022). However, such approaches may only be feasible in some training settings and not necessarily easily administered in competition. Further empirical research is needed to determine whether mental fatigue is directly related to injury risk and how such strategies may potentially limit risk of injury.

In the current study, participants provided varied perspectives on whether mental fatigue differs between sex (assigned at birth, male and female) and the potential resulting injury risk. There was no discussion on direct links, and perceived risks of injuries were indirect, such as general conditioning, biological differences and cognitive capacity. Hardaker et al. (2024) suggest that biological sex (male or female at birth) impacts risk of specific injury with female athletes showing a higher risk for knee (rate ratio [RR] = 2.7) and foot/ankle injuries (RR = 1.25), sports‐related concussions (RR = 1.3) and bone stress injuries (RR = 3.4). Conversely, male athletes had a 1.4–2.3 times increased risk of hip/groin injuries and a 2.4 times greater risk of hamstring injury than female athletes (Hardaker et al. 2024). Consistent with the present study, Hardaker et al. (2024) identify several potential reasons, such as differences in strength and biomechanics, but did not explore mental or cognitive aspects as contributing factors. The present findings suggest that while biological differences between sexes contribute to injury risk, the role of mental fatigue in this disparity is not clear.

Regarding gender and mental fatigue, limited research has investigated differences in mental fatigue between male and females in an athletic population. Díaz García et al. (2023) investigated the changes in mental fatigue during a padel competition and analysed reported mental fatigue between male and female athletes. The authors found no differences in reported mental fatigue (VAS) between genders. These findings in mental fatigue have also been replicated in healthy males and females (non‐athletes), with no significant differences in mental fatigue (VAS) following a mentally fatiguing task (Jaydari Fard and Lavender 2019). However, the findings in phase two of the present study suggest that participants perceive male and female athletes experience mental fatigue differently, though the impact on injury was uncertain.

Participants in phase one highlighted the broader impact of mental fatigue on athlete availability, suggesting that mental fatigue influences both preparation and the likelihood of illness. These results are consistent with research by Strand and Samuelson (2021), who surveyed collegiate athletes on physical and mental exhaustion and found that prolonged states of mental exhaustion may lead to burnout, with some participants reporting sickness. In phase two, participants predominantly perceived that mental fatigue may limit participation, although it was often contextualised alongside other factors.

None of the participants in this study consider mental fatigue in their training design with the specific intention of reducing injury. This reflects a more traditional focus on physical conditioning, where mental fatigue was managed incidentally (Russell et al., 2024). Despite most participants acknowledging an association between mental fatigue and injury, the lack of integrating mental fatigue into training is perhaps unsurprising, given that 44% of participants reported being ‘not at all’ aware of existing literature. Some participants did take steps to mitigate mental fatigue when planning training. Notably, one participant actively conducts tactical analysis 2 days before a game in an attempt to reduce mental fatigue on match day, demonstrating an example of practical recognition of mental fatigue's impact on athlete performance, even if not explicitly linked to injury prevention.

One major limitation of this study is that all survey respondents held the perception that mental fatigue and injury are linked. Additionally, there is potential for a response bias, as practitioners who chose to engage with the survey might have had a prior interest in the study's topics, despite the survey link being publicly accessible. A Hawthorne effect may also be present, with participants possibly responding in a way they thought aligned with the study's purpose. Future research could benefit from maximum variation sampling to provide a broader understanding of this association across different sports and populations. Nevertheless, our findings suggest that participants perceive an association between mental fatigue and injury, which may reflect a consensus within the field. Another limitation of this study is that participants were from a limited number of countries and potentially specific sports within those countries, which may restrict the generalisability of the findings to a broader population.

Participants emphasised the need for more research into objective measures of mental fatigue. Subsequently, a clearer understanding of the association between mental fatigue and injury risk is necessary, as highlighted by several participants, to justify its inclusion within training programmes. Future research could benefit from longitudinal study designs that collect mental fatigue data alongside injury data during training and competition to investigate potential associations. Further, future research should explore methods to systematically incorporate mental fatigue in training with the aim to reduce injury risk.

## 5 Conclusion

The primary findings of this novel study suggest that practitioners involved in injury management within high‐performance invasion sports perceive there to be a link between mental fatigue and injury risk. Participants suggested that noncontact injuries are more likely to occur under periods of elevated mental fatigue. Potential mechanisms proposed by practitioners to link mental fatigue and risk of injury include impaired motor control, poor biomechanics and reduced cognitive function. The challenge in distinguishing mental fatigue as a directly contributing factor to injury is acknowledged, given mental fatigue typically occurs alongside physical fatigue. Future research focused on the mechanisms linking mental fatigue demands to injury risk is required to empirically examine and determine the validity of the perceptions discussed in this study. Nevertheless, practitioners who perceive mental fatigue as a potential risk factor for injury might consider incorporating athlete management strategies, such as mental fatigue screening or implementing targeted training breaks.

## 6 Practical Applications

- Practitioners perceive that mental fatigue may increase the risk of injuries, in particular noncontact injuries, in high‐performance invasion sport athletes.
- Perceived indicators of mental fatigue which may relate to injury risk include deterioration in athlete posture and technique, decreased focus and poor decision‐making and behavioural changes such as disengagement.
- Practitioners could consider implementing strategies to reduce mental fatigue when indicators are observed, potentially mitigating injury risk. Approaches include brief rest breaks, caffeine‐maltodextrin mouth rinses or exposure to pleasant odours.

## Conflicts of Interest

The authors declare no conflicts of interest.

## Supporting information

Supporting Information S1
