# Supplementary material for: Practitioner Perspectives on the Association Between Mental Fatigue and Injury Risk in High‐Performance Sport: A Mixed Methods Study
Source: Eur J Sport Sci. 2025 Jul 29;25(8):e70028. doi: 10.1002/ejsc.70028 (PMC12307965; doi:10.1002/ejsc.70028)
Supplement: Supplementary file 1 — Supporting Information S1 [file EJSC-25-e70028-s001.docx]

**Appendix A.** Interview Schedule

**Q1 - CAN YOU PROVIDE AN OVERVIEW OF YOUR EXPERIENCE AND BACKGROUND IN HIGH-PERFORMANCE SPORTS AND INJURY PREVENTION OR MANAGEMENT?**

- **Q1.1** Can you share some specific examples of high-performance sports settings where you’ve worked?
- **Q1.2** Define athletes and sport worked with (prompt on sex of athletes, type of sport(s), level of competition(s).

**______________________________________________________________________________________**

**Q2 – MENTAL FATIGUE AND INJURY IN CONTEXT OF HIGH-PERFORMANCE SPORT?**

Just before we start, to ensure we are both clear on the research questions for consistency. I am just going to provide a couple definitions for you. Feel free to ask at any stage if you want me to repeat them too.

**Mental fatigue** (also known as cognitive fatigue) is a psychobiological state caused by prolonged periods of demanding cognitive activity, and has been shown to have negative effects on physical, technical, tactical, psychological and psychomotor aspects of sporting performance. Mental fatigue is indicated by changes in subjective, behavioural and/or (neuro)physiological changes.

**Injury** is tissue damage or other derangement of normal physical function due to the participation in sports, resulting from rapid or repetitive transfer of kinetic energy. Injuries can be broken down into, direct contact mechanisms, Indirect contact, non-contact mechanisms (sudden on-set) and Gradual-onset injuries, (which by their nature, are non-contact).

**Are you comfortable with those definitions? [Seek confirmation and/or clarify any questions]**

So, with those two definitions in mind;

- **Q2.1** Do you think mental fatigue affects an athlete's risk of injury?
- **Q2.2** Can you elaborate on the specific mechanisms through which you think mental fatigue influences athlete injury risk?
- **Q2.3** From your experiences, are there any indicators that you observe that tend to change when an athlete is mentally fatigued, potentially increasing the risk of injury?
  - If so, what are these and how do you measure them?

**______________________________________________________________________________________**

**Q3 – SPECIFIC INJURIES & THE IMPACT OF MENTAL FATIGUE?**

In relation to injury type, direct contact, in-direct contact, non-contact and gradual onset..

- **Q3.1** Do you think there are specific injuries that are more common when an athlete is mentally fatigued?
  - If yes, why?
  - Interviewer to ensure clarify on injury (and type), and context of athletes and sport.
- **Q3.2** Conversely, are there any specific injuries that seem to be less affected by mental fatigue, and if so, why do you think that is?
- Interviewer to ensure clarify on injury (and type), and context of athletes and sport.

**______________________________________________________________________________________**

**Q4 – POTENTIAL SEX DIFFERENCES**

Now we do have a question on the differences between sex. For the purpose of todays interview, we are referring to sex in regard to the physical sex characteristics recorded at birth – being male or female. But noting, this may be different to the gender of which they currently identify with today.

Do you think there are any differences between the potential association between mental fatigue and injury between male and female athletes?

- - If so, what / why?

**______________________________________________________________________________________**

**Q5 – CASE STUDY CONTEXT**

Now we have a specific case study question.

Do you have any specific notable examples of injuries encountered where you believe mental fatigue was a contributing factor?

- If so, can you talk me through it? describing the context, type of injury, and how mental fatigue played a role?
- Interviewer to ensure clarify on injury (and type), and context of athletes and sport.

**______________________________________________________________________________________**

**Q6 – ATHLETE AVAILIBILITY**

Now we have a question on athlete availability, which refers to the unrestricted ability for an athlete to participate in training or competition.

Do you think mental fatigue has an impact beyond musculoskeletal injury on athlete availability / unavailability.

- if so in what way?

**______________________________________________________________________________________**

**Q7 – MENTAL FATIGUE & DELIBERATE INJURY PREVENTION STRATEGIES**

- **Q7.1 -** Do you factor mental fatigue into your general injury prevention strategies / approaches?
- **No = Go to next Q.**
- **Q7.2 –** Can you provide an example of a specific strategy or protocol that you've found effective in mitigating injury risk when an athlete is mentally fatigued?
- **Q7.3 -** Do you think these strategies implemented intentionally to reduce injury risk in a MF state - are effective or ineffective?
- **Q7.4 -** Are there any challenges or obstacles you've faced in implementing these strategies, and how have you addressed them?

**______________________________________________________________________________________**

**Q8 – PREVENTION OF MENTAL FATIGUE & WITH INJURY PREVENTION IN-MIND**

- **Q8.1 -** Do you deliberately implement strategies to mitigate or limit mental fatigue, with the aim of reducing risk of injury in mind?
- **No = Go to next Q.**
- **Q8.2 -** Can you provide an example of a specific strategy or protocol that you've found effective in reducing mitigating mental fatigue with aim to reduce injury risk?
- **Q8.3 -** Are there any challenges or obstacles you've faced in implementing these strategies, and how have you addressed them?
- **Q8.4** – Even if you are not currently deliberately mitigating mental fatigue are there any factors, tasks, activities etc. that you think could be modified to prevent inducement of mental fatigue, with injury prevention in-mind?

**______________________________________________________________________________________**

**Q9 – OPEN QUESTIONS**

**Now we have a couple questions in relation to future research**

- **Q9.1 –** Do you have any questions in relation to the potential association between mental fatigue and injury or athlete availability, that if we could answer, would aid your practice?
- **Q9.2 -** Do you have a preference for how that information would be presented to you?
  - e.g. specific protocol to apply, general summary guidelines, case study examples?
- **Q9.3 –** What are the main current barriers you identify that could be improved to advance your knowledge and current practice around mental fatigue and injury?
- **Q9.4 –** How do you think specific roles within the high-performance team could better manage mental fatigue in relation to injury?
  - Reflect on your own and other roles.

**______________________________________________________________________________________**

**Q10 – OTHER INFORMATION**

Is there anything else you would like to add about your experiences or perceptions and opinions on mental fatigue in relation to injury and athlete availability that we have not yet explored in this discussion?

**END RECORDING OF SEMI-STRUCTURED INTERVIEW**
